# Carotid Atherosclerosis, Endothelial Disfunction, and Arterial Stiffness in Young and Middle-Aged Men with Coronary Artery Disease

**DOI:** 10.1155/2012/950130

**Published:** 2012-02-28

**Authors:** Sergey Kozlov, Tatyana Balachonova, Heda Machmudova, Marya Tripoten, Marina Andreevskaya, Anatoly Rogoza, Valery Kuharchuk

**Affiliations:** Institute of Cardiology, Cardiology Research Center, 3rd Cherepkovskaya street, 121552 Moscow, Russia

## Abstract

*Background*. To assess structural and functional status of the arteries in young and middle-aged men with recently developed CAD. *Methods and Results*. A total of 78 men aged 28 to 50 years underwent carotid ultrasound, endothelial function, and arterial stiffness measurements. Angiographically proven CAD was present in 49 patients. Carotid plaques were present in 45 (91.8%) patients with CAD and in 8 (27.6%) men without CAD (*P* < 0.001). Patients with CAD more often had multiple plaques (86.7% versus 13.8%, *P* < 0.001). The prevalence of carotid intima-media thickness (IMT) ≥0.9 mm and of abnormal brachial artery flow-mediated dilatation (FMD) was not differing in both groups. The mean aortic pulse wave velocity (PWV) was 6.8 ± 1.3 m/s for patients with CAD and 5.8 ± 0.9 m/s for subjects without CAD (*P* < 0.001). Aortic PWV above the 75th percentile of the “normal” samples was found in 26 (53.1%) patients with CAD and in 7 (24.1%) subjects without CAD (*P* = 0.02). Aortic PWV was related to the severity of CAD. *Conclusions*. Carotid plaques and increased aortic PWV may be more powerful predictors of the occurrence of CAD in young and middle-aged men, than the presence of increased carotid IMT and abnormal brachial artery FMD.

## 1. Introduction

Current guidelines for the primary prevention of coronary artery disease (CAD) recommend initial assessment and risk stratification with global risk assessment tools such as the Framingham risk score (FRS), which is based on traditional risk factors [1]. Recognizing that risk assessment strategies may inadequately assess cardiovascular disease (CVD) risk, noninvasive measures of atherosclerosis have emerged as adjuncts to traditional cardiovascular disease risk factors in an attempt to refine risk stratification and the need for more aggressive preventive strategies [2]. Arterial ultrasonography and tonometry are attractive methods for detecting subclinical atherosclerosis because they are noninvasive and relatively inexpensive. Testing for alterations in arterial function and structure with these methods includes measurement of carotid intima-media thickness (IMT) and plaque, measurement of brachial artery flow-mediated dilatation (FMD), measurement of ankle-arm index pressure (AAI), and determination of aortic pulse wave velocity (PWV) [3]. Prospective population-based studies have shown that diagnosis of subclinical atherosclerosis in asymptomatic patients may be considered as the screening test for predicting subsequent CAD events [4].

The purpose of the present study was to assess structural and functional status of the arteries in young and middle-aged men with recently developed CAD, who have had relatively low CVD risk before appearances of clinical manifestations of the disease.

## 2. Materials and Methods

A total of 49 consecutive male patients if they were no more than 50 years old who had been referred to our department between January 2009 and December 2010 for assessment of CAD was included in the present study. The inclusion criteria were angiographically documented: (≥50% stenosis of one or more of the major epicardial coronary arteries) CAD, recent (less than 6 months before the investigation) appearance of the first clinical features of CAD, and a stable condition. The results of the assessment of structural and functional status of the arteries were compared with those of 29 practically healthy control male subjects matched for age who were recruited from hospital staff and their relatives. All of them had normal ECG patterns, normal findings of ultrasound examination of the heart, and negative treadmill test results. Those with grade 2 and 3 arterial hypertension, diabetes mellitus, or LDL cholesterol (LDL-C) >4.5 mmol/L were excluded from the study. All patients underwent a measurement of carotid IMT and plaques, measurement of brachial artery FMD, and ultrasonography determination of aortic PWV.

### 2.1. Ultrasound Measurements

High-resolution B-mode ultrasonography was performed with a 5–9 MHz linear-type probe (PHILIPS iU22 ultrasound system, Philips Inc). Mean-IMT measurements were made in accordance with a Consensus Statement from the American Society of Echocardiography Carotid Intima-Media Thickness Task Force [5]. The individual value of mean-IMT was the mean of mean-IMTs of the right and left carotid arteries. Maximum-IMT was defined as the highest distance between the far wall lumen-intima and media-adventitia ultrasound interfaces taken bilaterally in the 1 cm segment proximal to the bifurcation. The individual value of maximum-IMT was the maximum of mean-IMTs of the right and the left carotid arteries. The measurement of carotid IMT and plaque was made by the same skilled operator. The intraobserver coefficient of variation for repeated measures of carotid IMT in our laboratory is 5.3%. IMT considered being normal if its value was <0.9 mm. The presence of atherosclerotic plaque was estimated at 6 sites of carotid pool: the whole length of both common carotid arteries (CCAs), both bifurcations, and both internal carotid arteries (ICAs). Plaque was defined as a focal structure that encroached into the arterial lumen demonstrated a thickness of ≥1.5 mm as measured from the media-adventitia interface to the intima-lumen interface [6]. Percent area stenosis was defined in the region of maximum plaque obstruction in the artery in the transverse view and was obtained from measurements of the residual lumen area and the original area.

Brachial FMD was assessed in a subject's right arm in the recumbent position after a 15 min equilibration period in a temperature-controlled room (22 to 25°C). Brachial artery diameter (BAD) was measured by B-mode ultrasound images at end diastole. The artery was longitudinally imaged ~5 cm proximal to the antecubital crease, where the clearest image was obtained and BAD was measured. After the baseline resting scan, a pneumatic tourniquet placed at the level of the mid forearm (proximal to the target artery) was inflated until no blood flow was detected through the brachial artery with the Doppler probe, and this pressure was held for 5 min. Increased flow was then induced with sudden cuff deflation, and a continuous scan was performed for 1 min. For the reactive hyperemia scan, BAD measurements were taken 45 to 60 s after cuff deflation. FMD was calculated from the diameters as (reactive hyperemia − baseline)/baseline × 100%. The measurement of brachial artery FMD was made by the same skilled operator. The intraobserver coefficient of variation for repeated measures of FMD in our laboratory is 9.2%. According to our previous results of FMD measurement in 46 healthy individuals, the values of FMD ranged from 4.1 to 22.2% (median, 8.1%), and none of them had FMD < 4.0%, so we set the “severe impaired” cutoff value of FMD at <4.0%. The values of FMD in the lower two tertiles (4–8%) were considered to be “impaired”, and we considered FMD to be “normal” when vasodilatory response of the brachial artery was >8%.

Aortic PWV was measured from serial Doppler flow signals obtained from the proximal (at the base of the sternum) and distal (above the navel) aorta with Vivid 7 Cardiovascular Ultrasound System (GE Healthcare). Digitized data were recorded by custom programming for subsequent analysis. A minimum of 10 beats was averaged for each recording site using the QRS for synchronization. Three separate runs were recorded for each participant, and all usable runs were averaged. The distance between the jugular notch and the navel sampling sites was measured above the surface of the body with a metal tape measure. The associated distance was divided by the difference in the time from the QRS to the onset of the pulsed-wave Doppler envelope in proximal and distal sites to produce aortic PWV. The measurement of aortic PWV was made by the same skilled operator. The intraobserver coefficient of variation for repeated measures of aortic PWV in our laboratory is 7.6%.

### 2.2. Statistical Analysis

Student's *t*-test for continuous data and *χ* ^2^ test for categorical data were used to compare the distribution of baseline characteristics between patients with and without CAD. The differences between groups with one-, two-, and three-vessel disease were analyzed using Kruskal-Wallis analysis of variance (ANOVA) by ranks. The association of CAD risk factors with premature CAD was examined with logistic regression. *P* < 0.05 was considered statistically significant.

## 3. Results

Clinical characteristics of the study patients are summarized in Table 1. Patients with CAD when compared with subjects without CAD more often had a family history (FamHx) of premature CAD (*P* < 0.01), low levels of HDL cholesterol (HDL-C) (*P* = 0.04), and more often were smokers (*P* < 0.01). As for other characteristics, there was no significant difference between both groups. The OR with a FamHx of premature CHD for the presence of CAD was 1.71 (95% Cl, 1.32 to 2.21), with smoking was 2.02 (95% Cl, 1.13 to 3.62), and with low HDL-C was 1.52 (95% Cl, 1.04 to 2.12).

### 3.1. Carotid Ultrasound

Average carotid IMT was significantly higher in CAD (0.88 ± 0.23 mm) relative to non-CAD patients (0.76 ± 0.18 mm, *P* = 0.01) (Table 2). Eighteen (36.7%) out of 49 patients with CAD and five (17.2%) out of 29 patients in control group had carotid IMT ≥0.9 mm, but this difference was statistically insignificant (*P* = 0.08). There was no correlation between carotid IMT and the severity of CAD as assessed by coronary angiography.

Among patients with CAD, 91.8% of the participants had at least 1 carotid plaque, 86.7% had more than 1 plaque, and one (2%) patient had significant (60%) stenosis (Table 2). Among patients without CAD, 27.6% of the participants had carotid plaques which were notably lower than in CAD patients (*P* < 0.001), and none of the control subjects had stenosis of 60% or more. Four (13.8%) out of 29 patients had more than 1 plaque that was lower than in patients with CAD (*P* < 0.001) The severity of CAD did not significantly correlate with the presence of carotid plaques.

### 3.2. Endothelial Function

Mean systolic blood pressure during measuring of FMD in patients with CAD (117 ± 7 mm Hg) and in patients of control group (115 ± 7 mm Hg) did not differ significantly. Mean brachial artery FMD was 4.5 ± 2.8% in patients with CAD (Table 2). Regarding the severity of disturbances, 46.9% of the participants had severe abnormal (<4%) FMD, 42.9% had abnormal (4–8%) FMD, and 10.2% had no abnormalities of FMD. In patients without CAD, mean brachial artery FMD was 5.8 ± 2.2% which was lower than in patients with CAD (*P* = 0.03). 34.5% of the participants had severe abnormal FMD, 48.3% had abnormal FMD, and 17.2% had no abnormalities of FMD. Frequency of abnormal FMD values did not differ between the two groups. We did not find association between the severity of CAD and the presence of abnormal FMD.

### 3.3. Arterial Stiffness

**Figure 1:**
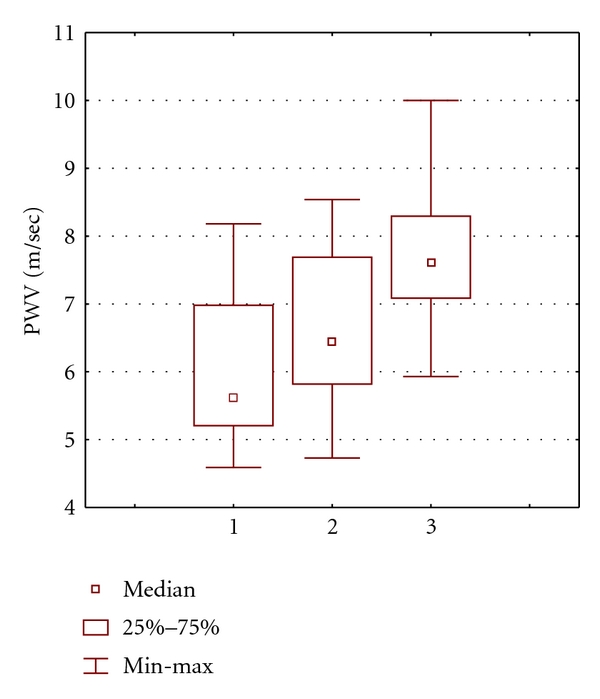
Aortic PWV in CAD patients. (1) one-vessel disease, (2) two-vessel disease, (3) triple-vessel disease. *P* < 0.01 (Kruskal-Wallis ANOVA by Ranks).

Mean systolic blood pressure during measuring of PWV in patients with CAD (120 ± 6 mm Hg) and in patients of control group (118 ± 5 mm Hg) did not differ significantly. Mean aortic PWV was 5.8 ± 0.9 m/s (median, 5.6 m/s; range, 4.4–7.8 m/s) in control subjects (Table 2). The upper quartile (above which PWV was deemed “above normal”) was 6.5 m/s. Mean aortic PWV was 6.8 ± 1.3 m/s (median, 6.9 m/s; range, 4.6–10.0 m/s) in CAD patients which was higher than in patients without CAD (*P* < 0.001). A higher proportion of patients with CAD had PWV ≥ 6.5 m/s (53.1% versus 24.1%; *P* = 0.02). Mean aortic PWV was 6.0 ± 1.1 m/s, 6.7 ± 1.1 m/s and 7.7 ± 1.2 m/s in patients with one-vessel, two-vessel, and three-vessel disease, accordingly. These differences were statistically significant (Figure 1).

Adjusted for FamHx of premature CAD, smoking, and low level of HDL-C, the existence of carotid plaques [5.3 (95% Cl, 2.14 to 13.12)] and increased aortic PWV [1.54 (95% Cl, 1.11 to 2.15)] were still related to the presence of CAD.

## 4. Discussion

Most information concerning prognostic value of noninvasive measures of arterial function and structure have been derived from the study of older populations. Therefore, there is a need to identify younger subjects at risk for CVD so that preventive measures may be instituted before occlusive vascular disease occurs. Patients with premature CAD included in this study were 26–50 years old, none of them had diabetes mellitus, 55% had normal values of LDL-C, and only 2 patients had mild arterial hypertension. First clinical CAD event, which was myocardial infarction in most (73%) patients, occurred without prior warning. 96% of patients may be considered at low and intermediate risk for future cardiovascular events when assessed with Framingham Risk Score method the day before first clinical manifestations of CAD. Patients with CAD included in this study when compared with subjects without CAD more often had a FamHx of premature CAD. It seems that addition of FamHx of premature CAD to global CVD risk models may improve prediction for future cardiovascular events in young and middle-aged patients. Potential value of inclusion FamHx of premature CAD to global CVD risk assessment has been demonstrated in several studies [7, 8].

Young and middle-aged men with a FamHx of premature CAD are candidates for quantification of subclinical atherosclerosis by noninvasive measures [9]. There is a lot of different noninvasive tests for diagnosis of subclinical atherosclerosis. The implementation of subclinical atherosclerosis testing in the risk management of patients is dependent on a better knowledge of the comparative prognostic performance of currently available tests. Among them vascular ultrasonography and tonometry are promising test modalities for assessment of arterial function and structure in asymptomatic subjects because they are noninvasive and relatively inexpensive. The prognostic value of each of them has been shown in prospective studies [4, 10], but the different types of subclinical atherosclerosis tests have a different prognostic performance [11].

The present study demonstrates that young and middle-aged men with premature CAD when compared with subjects without CAD more often have atherosclerotic plaques in the extracranial carotid arteries. Also patients with CAD more often have more than 1 plaque. This is concordant with previous studies, which have shown association between the presences of carotid plaques and CAD incidence [4, 12, 13]. 13221 low-risk, healthy, asymptomatic individuals were included in a 10-year prospective, followup-based CAFES-CAVE study [13]. The aim of the study was to assess the relation between subclinical arteriosclerotic lesions at the carotid and femoral arteries identified by B-mode ultrasound and the occurrence of future CVD events. Four classes were considered at inclusion (I: normal wall, II: wall thickening, III: nonstenosing plaques, IV: stenosing plaques). In 10 years, there were 0.1% events in class I, 8.6% events in class II, 39% events in class III, and 81% events in class IV. The increased event rates in classes III and IV were significant in comparison with I and II.

Patients with CAD included in this study had greater mean value of IMT. However, the difference between the occurrences of increased (≥0.9 mm) IMT values in both groups was statistically insignificant. Analysis of prospective epidemiological data in the general population was performed by Simon et al. [14] to determine the association of carotid IMT with CVD risk. They revealed that carotid IMT is an independent but relatively modest (as judged by absolute risk) predictor of CAD, carotid IMT adds little to the CHD prediction by risk factors, and plaque may be more representative of atherosclerosis than carotid IMT.

In our study, mean brachial artery FMD was lower in patients with CAD when compared with subjects without CAD, but the frequency of impaired FMD was the same in both groups. The assessment of FMD as a noninvasive approach to examine vasodilator function has been used to compare groups of subjects and to evaluate the impact of interventions within individuals. Data about the incremental prognostic value of FMD in asymptomatic subjects are limited. Anderson et al. [10] prospectively examined 1574 men (age, 49.4 years) free of vascular disease. These subjects had low median FRS (7.9%), and FMD was not associated with subsequent cardiovascular events (hazard ratio, 0.92; *P* = 0.54). Contrary to these findings, Yeboah et al. [15] found that brachial FMD is a predictor of incident cardiovascular events in population-based adults. Also the addition of FMD to the FRS did not improve discrimination of subjects at risk of CVD events in receiver operating characteristic analysis; it improved the classification of subjects as low, intermediate, and high CVD risk compared with the FRS. Despite FMD widespread adoption, there is considerable variability between studies with respect to the protocols applied, methods of analysis, and interpretation of results. Moreover, differences in methodological approaches have important impacts on the response magnitude, can result in spurious data interpretation, and limit the comparability of outcomes between studies [16].

According to our data, patients with CAD had higher mean aortic PWV. Significantly higher proportion of patients with CAD had PWV above “normal” (≥6.5 m/s) value. Moreover, there were association between the severity of CAD and the value of aortic PWV. The patients with CAD more often had therapy with ACE-inhibitors and statins, which may positively affect FMD and PWV. But in spite of this possible positive influence, patients with CAD more often had increased aortic PWV. There is evidence that increased aortic PWV is a predictor of subsequent CVD events in a general population [17]. PWV is usually measured by the foot-to-foot velocity method from various waveforms [18]. There is a variety of different waveforms including pressure, distension, and Doppler. The method we used in our study to calculate aortic PWV is based on Doppler probes. Whether it has any advantage as compared to more traditional methods based on pressure sensors remains to be seen, and normal values will need to be established for wider introduction of this method in clinical practice.

## 5. Conclusions

In summary, the results of the present study demonstrate that young and middle-aged men with recently developed CAD more often have structural and functional status abnormalities of the arteries, such as multiple carotid plaques and increased aortic PWV. These abnormalities may be more powerful predictors of the occurrence of CAD in young and middle-aged men, than the presence of increased carotid IMT and abnormal brachial artery FMD. These findings support the concept that assessment of structural and functional status of the arteries may play a role as an additional strategy to identify patients who would benefit from aggressive preventive measures.

## Figures and Tables

**Table 1:** Clinical characteristic.

	CAD (*n* = 49)	No CAD (*n* = 29)
Age (years)	43 ± 5	43 ± 6
History of myocardial infarction (%)	41 (80%)	—
Body mass index (kg/m^2^)	26.8 ± 3.5	26.4 ± 3.7
Hypertension (%)	2 (4%)	5 (17%)
Total cholesterol (mmol/L)	5,1 ± 1,0	5,1 ± 0,7
LDL-cholesterol (mmol/L)	3,3 ± 0,9	3,3 ± 0,6
HDL-cholesterol (mmol/L)	1,0 ± 0,2	1,1 ± 0,2
HDL-cholesterol <1.03 mmol/L (%)	28 (57%)**	9 (31%)
Triglycerides (mmol/L)	1,8 ± 0,6	1,4 ± 0,8
Currently smokers (%)	42 (86%)*	15 (52%)
Family history of CAD	14 (29%)*	1 (3%)
Angiographic characteristics		
One-vessel disease (%)	17 (35%)	—
Two-vessel disease (%)	17 (35%)	—
Triple-vessel disease (%)	15 (30%)	—

*P < 0.01; **P = 0,04 for difference compared with no CAD.

CAD: coronary artery disease.

**Table 2:** Structural and functional status of the arteries in young and middle-aged men with recently developed CAD.

Variable	CAD (*n* = 49)	No CAD (*n* = 29)	*P*
Carotid IMT (mm)	0.88 ± 0.23	0.76 ± 0.18	0.01
≥0.9 mm (%)	18 (36.7%)	5 (17.2%)	NS
Carotid plaques (%)	45 (91.8%)	8 (27.6%)	<0.001
More than 1 plaque (%)	39 (86.7%)	4 (13.8%)	<0.001
Brachial artery FMD (%)	4.5 ± 2.8	5.8 ± 2.2	0.03
>8% (%)	5 (10.2%)	5 (17.2%)	NS
Aortic PWV	6.8 ± 1.3	5.8 ± 0,9	<0/001
≥6,5 m/s	26 (53.1%)	7 (24.1%)	0.02

CAD, coronary artery disease; IMT, intima-media thickness; FMD, flow-mediated dilatation; PWV, pulse wave velocity.
